# Recent Advanced Diagnostic Aids in Orthodontics

**DOI:** 10.7759/cureus.31921

**Published:** 2022-11-26

**Authors:** Shalabh Baxi, Kiran Shadani, Rituraj Kesri, Ankita Ukey, Chirag Joshi, Harsha Hardiya

**Affiliations:** 1 Department of Orthodontics, Government Dental College, Raipur, IND; 2 Department of Orthodontics and Dentofacial Orthopaedics, Consultant Orthodontist, Raipur, IND; 3 Department of Pedodontics and Preventive Dentistry, Maitri College of Dentistry and Research Centre, Durg, IND; 4 Department of Oral and Maxillofacial Pathology, Rungta College of Dental Sciences and Research, Bhilai, IND; 5 Department of Prosthodontics, Hukumchand Lal Hospital, Indore, IND

**Keywords:** orthodontics, nanotechnology, latest technologies, 3d printing, digital imaging, aligners, advancements

## Abstract

Everyone aspires to have a youthful appearance, complete with a beaming grin. By treating skeletal and dental malocclusions that interfere with facial aesthetics, orthodontics helps patients attain a beautiful face and a smile they will be proud of. The diagnosis of the underlying illness or condition serves as the basis for all medical, dental, and surgical operations. Dental professionals all across the world have reaped the benefits of a major scientific advance in recent years. Many measurements may now be seen and quantified more easily using computer-assisted cephalometry. The accuracy and high quality of all dental materials have been enhanced thanks to computer-aided design and computer-aided manufacture. There have been several developments in the realm of orthodontics. The growing use of technology in recent years has transformed every sector, including medicine and dentistry. From Angle's time to today's nanorobotic age, orthodontic ideas, biomaterials, and technology have evolved greatly. It has been extensively utilized for diagnosis, treatment planning, three-dimensional printing, appliance systems, digital storage, integration, and data retrieval. The technology used in orthodontics is always getting better, and this article aims to give an overview of the most recent changes.

## Introduction and background

New diagnostic methods have made orthodontic diagnosis and treatment planning more important in our litigious society. It is crucial to maintain precise records of treatment progress since a poor record might suggest a low degree of orthodontic therapy [1]. For orthodontic records, a diagnostic report accompanied by study models, radiographs, and photographs is necessary to establish the case's state before treatment and to chronicle the course of treatment. Making an accurate diagnosis is made much easier with the use of dental records that are issue-oriented. The morphologically oriented diagnostic approaches have been greatly improved. The orthodontist's role is to put the human craniofacial complex back together [2]. This project's goal is to rearrange everything in a way that is both useful and visually appealing. Obviously, this approach must take into consideration the connections between the three specific axes. Despite the fact that orthodontic treatment impacts the patient in all three dimensions, many of the diagnostic instruments now in use only depict the patient in two dimensions. The aims of orthodontic therapy (function and aesthetics) are changing. Because of the advancement of digital technologies in private practice, an enhanced three-dimensional (3D) diagnostic and treatment planning technique has been established. 3D imaging in orthodontics makes it feasible to diagnose and arrange therapy in advance, as well as evaluate dentoskeletal relationships and facial aesthetics thereafter. It is also possible to use 3D models in orthodontics to manufacture customized archwires for research and medical-legal aims [3].

The advent of personal computers in the 1990s had a profound impact on the field of orthodontics. In practice management, digital orthodontics has ushered in a new era. Orthodontic practice in the 1970s and 1980s contrasts sharply with the innovative techniques of the new century. CT and 3D reconstruction in the 1970s revolutionized diagnostic radiology since cross-sectional imaging was now feasible. As techniques for 3D reconstruction improved during the 1980s, it became a common practice for radiologists and surgeons to employ 3D CT scans of regular two-dimensional (2D) CT data to produce pictures of complicated anatomic structures such as the skull for diagnostic purposes. Orthodontists began to rely on computers for diagnostic and treatment planning as they grew more dynamic and dependable. A professional orthodontist with knowledge of the biological and biomechanical knowledge base is still the most important component in successful orthodontic therapy. Appliances and aligners made with computer-aided design (CAD)/computer-aided manufacture (CAM) technology are also accessible. Using 3D printing, a wide range of objects may be created with incredible accuracy. Orthodontists have tested and documented the use of technology to make dental models, removable appliances, customized brackets and archwires, and occlusal splints [2]. The T-scan consists of a handle assembly, a big and small sensor and support, computer software, and a printer. In orthodontics, T-use scans are used to document occlusion prior to treatment and monitor changes over time to determine what is causing lateral interference. We must identify early and strong forces so that we can swiftly redistribute them, and make sure the occlusion is appropriate both visually and practically. To stop people from developing malocclusion later in life and obtain stable, long-lasting results, CAD is used to quickly create 3D models, which are then built up layer by layer according to the 3D input. There are various uses for nanotechnology in orthodontics, including nanocoatings on wires and nanoparticles in orthodontic adhesives, to name just a few of them. Using microsensor technology to track how portable appliances are used is also a new way to do things.

The goal of this study was to look at the most up-to-date diagnostic tools for planning orthodontic care.

## Review

Latest imaging techniques

Cone Beam Computed Tomography

Many 2D snapshot photos are taken at predetermined angles when the equipment rotates around the X-ray source/sensor unit during a cone beam computed tomography (CBCT) scan, which was launched in dental radiography in 1998 [2]. In comparison to traditional CT fan-shaped devices, the cone beam delivers a more concentrated beam with much less scatter radiation. CBCT has a lower cost, smaller size, a custom-built exposure chamber (head) that decreases radiation exposure, and pictures that are equivalent to standard CT and may be shown as a whole head view, as a skull view, or as regional components [4,5].

Clinical applications in orthodontics: Orthodontists now have access to a number of radiographic images that were previously impossible to obtain using conventional radiography techniques [6]. CBCT has the following applications in orthodontics: impacted teeth, oral anomalies, airway examination, alveolar bone height and volume evaluation, temporomandibular joint (TMJ) morphology, skeletal views, face analysis, and 3D study of dentition.

Radiation exposure: There are four times fewer radiation doses than normal CT scans with CBCT. The amount of radiation emitted is determined by the parameters entered (Kvp and mA). Inferior mA and collimation settings may limit radiation exposure to the patient; however, this results in lower picture quality than utilizing higher mA and collimation settings. The effective exposure of the patient to a resulting CBCT ranges from 45 Sv to 650 Sv. The ALARA (as low as reasonably achievable) concept advocates the adoption of procedures that minimize radiation exposure during dental radiography. This comprises taking radiographs based on the needs of the patient, using the fastest film suitable for the diagnostic work, collimating the beam size as close as feasible to the film size, and shielding the patient's body from radiation using lead aprons and thyroid shields.

Digital Cephalometry

Standardizing radiographic imaging of the skull was first proposed by Pacini in 1922, but his approach was crude. His recommendations included a film cassette fastened to the patient's head with gauze bandages and put at a fixed distance of 2 meters from the X-ray source. Later, in 1931, Broadbent and Hoffarth of the United States and Germany published their methods for obtaining consistent lateral head radiographs at the same time. The lateral cephalogram may provide information about a patient's dental, skeletal, and soft tissue morphology, as well as the linkages between these components [7]. To determine the morphology and development of the craniofacial complex, identify anomalies, anticipate future connections, and plan orthodontic therapy, cephalometry is a vital instrument. Researchers may measure the craniofacial characteristics of individuals and groups by using lateral cephalograms (LCGs), differentiating between what is considered normal and what is not, analyzing changes in pattern over time in populations by comparing treated samples to untreated ones, and defining populations as homogenous or heterogeneous. Digitized cephalometric imaging has recently become a realistic alternative due to the introduction of low-cost radiography (extraoral) and an increase in the use of computers in orthodontics. Orthodontics is seeing a paradigm change from commonly used film-based cephalometry to digital cephalometry. The evolution of digital cephalograms is similar to that of digital radiography, which has already been covered in detail. Liu et al. examined the accuracy of computerized landmark recognition using several angular and linear measurements [8]. For this reason, they concluded that more investigations are required to verify the accuracy of computerized landmark identification. Geelen et al. [9] wanted to determine whether or not cephalometric landmarks could be successfully reproduced on standard film, hard copy, and monitor pictures manufactured using the storage phosphor method. They concluded that there was no clinically relevant difference between the different techniques of landmark recognition. With so many options, it may be difficult for doctors to decide which one to use. Fortunately, there is a slew of software programs available to help them make that decision. Computerized digitizing methods such as Digiceph, developed at Indian Institutes of Technology Delhi's Center for Bio-Medical Engineering and the Department of Dental Surgery at All India Institute of Medical Sciences, as well as 13 cephalometric analyses, have all been created in India.

Digigraph

When it comes to orthodontic diagnostics, Kevin HY Mok and Michael S Cook [10] discussed the usage of digigraph. It is a method of digitally preserving sound. This technology aids in the measurement of linear distances. They contribute to reducing radiation exposure from patient diagnostics using lateral cephalometric tracings. After the plaster cast is digitized, the mesiodistal breadth of teeth is measured. Digital handpieces were used to collect mesiodistal measurements. The process is as follows: while pressing the handpiece's button, place the tip of the handpiece on the selected landmark for the scan to be performed.

Digital Imaging and Communications in Medicine (DICOM)

Together, the American College of Radiology (ACR) and the National Electrical Manufacturers Association (NEMA) developed a standard method for transferring medical images and the data that goes along with those images. In addition to photos, DICOM created information that was standard not just for patients, but also for students, reports, and other groupings of data. Because of DICOM, picture archiving and transmission technologies have grown in popularity (version 3.0). Its primary purpose is to help in the diagnosis and planning of treatment and to provide a 3D record of the original malocclusion, at any stages of correction, and the final treatment outcome [11,12]. It is important for an orthodontist to use study models since they are cast in plaster or stone, but they have a number of restrictions in terms of storage and retrieval, diagnostic flexibility, and transferability. Digital study models may be created in two ways: damaged imaging detains a portion of the cast while it is being photographed and analyzed in non-destructive imaging, and light sources like lasers, X-rays, and structured light are used to create a picture without damaging the original cast material. Among the leading manufacturers of high-quality 3D models, OrthoCad (Cadent, Fairview, NJ) [12,13] and GeoDigm (GeoDigm Corporation, Chanhassen, MN) [14] are deserving of special attention.

OrthoCad

About 10% of American and Canadian orthodontists [12] make use of OrthoCad's digital study model collection, evaluation, and archiving technology. Cadent is the company behind this piece of software (computer-aided dentistry). An optical scanner is used to capture the model's picture from a plaster equivalent. Using the patent OrthoCad software user interface, they are then shown to the orthodontist, who may manipulate the models in virtual space as well as gather data using a variety of diagnostic instruments. An accurate bite record and high-quality impressions are a need. Imprints made from high-quality alginate, polyvinyl silicone, or polyether are acceptable [15]. Impressions that are dimensionally exact and stable are the desired end result. Alginate impressions are cleaned by wrapping them in a heated paper towel and then sealing them in a plastic bag to retain the moisture. For long-term storage and transit, polyether materials may be used once impressions have been sent. Plaster counterparts of the imprints are created and optically scanned into the OrthoCad computer system without destroying the original plaster counterparts. As a final step, the patient's 3D virtual models are saved to the computer. OrthoCad D3 browsers enable models to be seen from five distinct angles at the same time, eliminating the need to rotate [12,13]. There are two ways of looking at this color scheme: as an analogy to a wax bite, and as a representation of the tightness of contact points between maxillary and mandibular teeth. The occlusogram is altered by lateral or vertical jaw shifts [16]. In contrast to plaster models, digital models may be sectioned at any point in the sagittal or transverse planes, unlike plaster models. There is a chance that this may shed new light on skeletal and dental asymmetries and help pinpoint skeletal and dental midlines. Any part of the model may be precisely sized using the virtual caliper (0.1 mm). Once the arch shape and size are known, the disparity in floor space may be determined. Each patient's folder comprises the digital model, which includes detailed contact points and dimensions. In addition, they may be e-mailed to other dentists and medical professionals with comments and measurements attached to them.

Advantages: It is an easier and more efficient approach to measure and store data from a virtual model, and it can be retrieved and seen together with the patient's other clinical data at the chairside, along with digital pictures, X-rays, and clinical notes, in the patient's digital file. It may be shared with other healthcare workers through printouts or email attachments.

Disadvantages: Virtual models cannot be installed and described in terms of the TMJ capabilities of patients, despite the fact that the jaw arrangement device is near to that. They are pricey, averaging 36 dollars per model set and 55 dollars for shipping, as well as time-consuming. Most orthodontists only employ them in 10% of their practices since they are not legally recognized as advanced concentrate on models.

GeoDigm

Using a precision of 0.1 millimeters, GeoDigm e-models are created by scanning patients with a patented laser scanning technology. These scanners employ digital cameras to scan the cast and look for any distortions in a laser stripe that is projected onto the surface. To expose the cast for scanning, it is orientated in all four directions. Through the use of thousands of interconnected triangles, the 3D image is built up from these 3D vertices. Each triangle in the e-model is given a color based on its distance from a digital light source on the computer display when it is generated in software. In the end, you will have a 3D image that you can hold in your hand and alter in real time on your computer screen. It is then sent to GeoDigm, where an e-model is created from the impressions and bite registration, and the e-model is articulated. The model may be fetched from the main server. As an extra precaution, the GeoDigm server stores a duplicate of all data. Using models, the physician is able to move, rotate, or zoom in any direction or position. All of these functions may be performed by simply clicking and directing the mouse at a certain location. A 3D color-coded map of occlusal content between arches is provided by the color-bite mapping capabilities of the software. In addition, the clinician may adjust the rotational axis using an articulation function.

Radiovisiography

In digital radiography, the first system was launched. A system for imaging dental work with minimum exposure to radiation and many more benefits than traditional radiography. Without the need for a dark chamber, it was able to create immediate photographs [17].

Conventional Tomography

Hounsfield came up with this design in 1972. CT uses a computer to produce a picture. The 3D shape of an image may be achieved by the use of several slices of a picture. However, the CT's soft tissue contrast is not as effective as it may be [18].

Tuned Aperture CT

DA Tyndall, JB Ludlow, RL Webber, and RA Horton developed a new method for collecting 3D radiography data in their paper [19]. Transmission radiography is another name for it. Tuned aperture CT (TACT) pictures are created using a reference point and a large number of X-ray projections. They aid in the visualization of hard tissues in the mouth. TACT slices may be generated from any number of X-ray projections. For a projection to be accurate, it must include a reference point that is placed above the detection plane. One X-ray source may be utilized to make several X-ray projections by moving it around in space to create TACT slices. Caries periodontal and periapical diseases cannot be accurately diagnosed with these photos.

Multi-Detector CT

It is a kind of diagnostic CT imaging. Detector elements are arranged in a 2D array. As a result, several thin slices may be obtained and a quicker CT picture is formed. 3D pictures may be reskinned in many planes and panoramic views can be reconstructed using special techniques. It assesses pathologic abnormalities more precisely. As a result, it lowers patient motion artifacts owing to shorter capture times. It has greater soft tissue resolution with fewer artifacts and scattering radiation than CBCT. An oblique or curved picture plane may be created as well as any of the four previously mentioned ones [20,21].

Ultrasonography

Pressure fluctuations in the air against the eardrum cause sounds to be interpreted as such. Between 1500 and 20,000 cycles per second, these modifications occur (hertz, Hz). Ultrasound is defined as having a frequency higher than 20 kHz. Since its vibratory frequency exceeds the hearing range, it may be recognized from other mechanical waveforms. A vibratory frequency of 1-20 MHz is used in diagnostic ultrasound, which is a clinical application of ultrasound imaging and analysis [22]. Devices that convert sonic energy to electrical energy are used in sonography scanners, which create electrical impulses and employ high-frequency sound waves as a transducer. Because of this abrupt shift in vibration, the sound waves that are being delivered into the tissue under examination are caused by the scanner's electrical impulses realigning crystal dipoles in the electric field. Absorption, reflection, refraction, and diffusion all work together to reduce the ultrasound's power as it travels through and interacts with various types of tissue [23]. An electric signal is generated from the piezoelectric crystal when sonic waves are reflected to the transducer and amplified, processed, and then shown on a monitor. This arrangement uses an integrated transducer/receiver/transmitter device. Typical lateral resolution for high-resolution systems is 1 millimeter, with thin-film thicknesses of 0.5 millimeters or less. Modern methods enable us to analyze echoes at a fast enough pace to detect movement. This is known as real-time imaging [24].

Applications (in head and neck): Predominantly for lymph nodes, post-surgery images, and images of swelling and hematoma, submandibular salivary gland, eye, thyroid, and parotid glands. It exhibits soft tissue displacement beneath a denture by occlusion pressures and demonstrates masticatory mucosal thickness [25]. Many orthodontists are interested in how the tongue operates when swallowing. Perioral muscles contract little during deglutition, teeth are briefly in touch with each other during swallowing, and there is no tongue push or forward posture during deglutition in normal conditions. In earlier investigations, researchers have utilized a variety of approaches to measure tongue movements, including radio-cinematography, electromyography, and electromagnetic articulography. Normal tongue function cannot be examined with electropalatography or electromagnetic articulography because the receiver coils and wires attached to the palates and tongues of patients make swallowing difficult. Cinematography and CT that use X-rays have the disadvantage of exposing the patient to radiation. There is no way to investigate swallowing movements using MRI because of the high cost and long acquisition time [26]. As a non-invasive, time-saving, and cost-effective diagnostic tool, ultrasound is an excellent option. There have been a number of studies that have used ultrasonography to get a static image of the mouth cavity; for example, in the research of tongue morphology and the diagnosis of sialolithiasis, cysts, and tumors. A technique called dynamic ultrasonography has been reported on by professionals for years to follow the movement of the tongue underneath the conscious consciousness. There was a problem with previous dynamic ultrasound studies since they used direct transducer-skin coupling scanning to monitor tongue movements, which resulted in various artifacts causing inaccurate evaluations of tongue motions to be made. A cushion scanning method may be able to help with these issues. If an infrared camera, head support, and a cushion are all used in a scanning system, tongue dynamics may be reliably tracked. To research tongue morphology and tongue functions, such as swallowing and speaking, real-time B+M-mode ultrasonography with cushion scanning technique (CST) is presently the most often used tool for this purpose. 3D ultrasound imaging is a new technique that produces more detailed photographs of the fetus's face than earlier 2D imaging methods have managed to achieve. These benefits include being able to adjust planar views without worrying about fetal movement, determining the precise placement of planar pictures with respect to the surface facial image, and giving non-trained observers simple access to realistic 3D visuals. When it comes to diagnosing cleft lip and palate, 3D imaging has substantially greater sensitivity than 2D imaging.

SureSmile

Computer administration, 3D imaging of dentition, complicated 3D data processing, and robots have led to a novel treatment strategy [27]. Patient-centered practices give high-quality care with minimal pain, compliance demands, and chair time, and finish treatment on schedule. There are several benefits to using SureSmile (Dentsply Sirona, Charlotte, NC), including the reduction of treatment mistakes due to poor appliance management, picture capture, 3D visualization of diagnostic instruments, and improved communication between orthodontists and their patients via the use of precision appliances.

Clinical procedures: The oro-scanner (oro matrix) is a hand-held scanner that collects real-time, in vivo pictures of the patient's teeth [28]. A thin white film is applied to the teeth to prepare them for scanning, much like an articulating spot spray. To create a quick series of pictures, a perfectly designed grid is projected onto the teeth, which are illuminated with structured white light. As the handheld scanner is moved over the teeth, a video camera incorporated into the scanner's grip records photographs of the reflected dentition and the deformed grid. A rocking motion is used to move the scanner across the dentition to see all of the tooth surfaces, including the undercuts. It takes roughly 112 minutes for each arch to complete. It is possible to move the scanner from one chair to the next using a mobile care smile cart. Multiple and overlapped pictures are sent to the computer during the scanning step. Image processing and computer modeling are made possible thanks to advanced data registration and management approaches. Finally, a library of dental morphology is used to compare the teeth to those in the library. To improve models even more, missing data from the scan are filled in using data from the library. As part of the patient's electronic medical record, an entire mouth scan is obtained together with traditional pictures and X-rays. It is possible to move the teeth independently in 3D using software controls after the surgery is complete. A diagnosis, treatment plan, and simulation of outcomes are all possible with a program based on Microsoft Windows (Microsoft Corporation, Redmond, WA). Tooth movement that is not desired may be minimized, and errors in arch-wire selection may be eliminated, if possible. There may be a reduction in the number of bracket placement mistakes. Due to this method, bonding adhesive thickness mistakes are eliminated.

DentaScan

There are three planes of view for the maxilla and mandible in DentaScan (GE Healthcare, Chicago, IL): axial, panoramic, and cross-sectional. It is very beneficial in head and neck surgery because it improves the assessment of the osseous maxilla and mandible [29-31]. DentaScan examinations are painless and straightforward. It is recommended that the patient removes any jewelry, dentures, hair accessories, and hearing aids before the examination. High-resolution spiral CT is utilized in DentaScan to capture pictures for image analysis. The patient's jaw is held in place on a Styrofoam surface, coupled to the head holder of the CT scanner, such that the mandibular base is perpendicular to the horizontal axis. Radiation doses of 120 kVp and 120 Ma of X-ray energy are used in CT scans. These axial CT data are delivered to a workplace where DentaScan is utilized to reformat the images to produce panoramic and paraxial images on film. As a result, dental implants may be placed more precisely thanks to the information provided by DentaScan. A jaw fracture or cyst may also be detected with this technique. Aside from the higher radiation exposure and the high cost, it has a few drawbacks. Choosing the right patient for DentaScan therapy is thus critical [32,33].

Various scanners application in orthodontic treatment planning

T-scan

The T-scan consists of a handle assembly, a big and small sensor and support, computer software, and a printer. In orthodontics, T-use scans are used to document occlusion prior to treatment and monitor changes over time to determine what is causing lateral interference. We must identify early and strong forces so that we can swiftly redistribute them to make sure the occlusion is appropriate both visually and practically and to stop people from developing malocclusion later in life and obtain stable, long-lasting results [34].

3D Facial Scanners

Face scanners make it possible to get a 3D topography of the facial surface anatomy, recognize facial landmarks automatically, and analyze the symmetry and proportions of the face. For example, face scanners may be used to monitor growth and development, ethnicity and gender disparities, and identify key diagnostic traits in selected groups of people with craniofacial anomalies.

Stereophotogrammetry

Stereophotogrammetry uses triangulation and stereo camera pairs in stereo setups to measure the 3D distance to features on the face [2]. By 1967, it had already been suggested by Burke and Beard [35].

Advantages of 3D photogrammetry: In a matter of milliseconds, the camera can take a picture, which reduces motion distortions and is particularly beneficial for babies. It is possible to check the image quality right away. The picture may be viewed and edited using a variety of applications. Facilitate the recognition of recognizable structures. Anthropometric linear, angular, and volumetric measures may all be computed using it. Limited availability, expensive, and time-consuming are some of the drawbacks of 3D photogrammetry. Faces with shiny, shadowy, or translucent features are difficult to capture, making them tough to analyze. It is incapable to compute interactive landmarks.

Moiré Topography

There are no needles or any other intrusive methods used in this system, and it uses only vision-based imaging. Based on the fringes and fringe intervals of the contours, Moiré topography provides 3D information [3]. Ray optics is used to get the fringes' depth and excellent precision is attained with simple equipment. Sharp characteristics on a surface make it tough to record. Smoothly shaped faces provide better outcomes.

Digital Storage

Digital imprint methods make it simpler to create digital study models. Efficient use of electronic systems reduces the quantity of physical storage required, the number of lost or damaged models, storage challenges associated with big study models, and transportation concerns. These are all issues that might be relevant in the context of audit and research. Many procedures benefit greatly from the online transmission of research models to labs for appliance manufacturing, which reduces transportation expenses and improves efficiency [36].

Virtual Orthodontic Patient

3D virtual orthodontic patients may now be created with the use of 3D imagery and four-dimensional face dynamics. Many soft and hard tissue investigations will be possible thanks to the notion of virtual orthodontic patients. Improved masticatory system and tooth movement biomechanics, as orthopedic and orthodontic knowledge [37], will be the result of these studies.

Prediction Imaging Software

A variety of software systems may be used to forecast the outcomes of orthognathic surgery, either alone or in conjunction with video pictures. Digital hard and soft tissue profiles may be manipulated using various software systems that enable doctors to create a pretreatment picture that can be used to create a therapy simulation. A few of the 3D forecasting technologies now available are surface scan/CBCT, 3D CT, and 3D MRI.

3D Printing

3D printing was invented in 1990 by Wilfried Vancraen, chief executive officer and director of Materialise NV (Leuven, Belgium) [38], the first Benelux rapid prototyping company. Using 3D printing, virtual models may be printed into 3D items, such as prototypes and manufacturing components.

Rapid Prototyping

Traditional layer-by-layer construction, known as rapid prototyping (RP), is a method for rapidly creating 3D models using CAD. In November 1987, a firm named 3D Systems, Inc. unveiled the first commercial RP process at the Autofact Exhibition in Detroit, USA. By first creating a solid 3D CAD model of an item and then breaking it down into cross-sectional layers and numerical data in the form of virtual trajectories; this technique is known as "layered manufacturing" or "solid free-form fabrication." Prototypes may be built in an automated fabrication machine using material additive methods that are guided by a computer.

Types of rapid prototyping: Fused testimonies, selective laser softening, specialized laser sintering, stereolithography, production of laminated objects by the use of inkjet printing, electron beam melting (EBM), and digital light processing (DLP).

Latest updates in appliances used in orthodontics

Align® Technology

The Invisalign braces for straightening teeth were created in the United States in 1998 by Align® Technology, Inc. Straightening teeth into a perfect occlusion is achieved by using thin, transparent, overlay sequential appliances. Treatment starts with a diagnostic and plan from the orthodontist. It is then delivered to Align® Technology together with the diagnostic and treatment plan as well as images of the patient and occlusal bite registration. 3D models may be created from the collected data using "destructive scanning" devices. From the 3D data, a 3D model is created. There are many phases to the therapy process, which go from the current state to the intended outcome. Orthodontist approval is required before photosensitive thermoplastic dental models can be made for each step of treatment. As a result, a series of transparent Invisalign aligners may be made. The patient is told to wear each set of aligners for roughly a week and a half before moving on to the next. These systems do not allow for any kind of orthopedic adjustments. While in Invisalign therapy, teeth do not continue to erupt and major alterations in the arch do not occur, and root location is not taken into consideration after treatment if a tooth's morphology changes as a result of restorations or composite build-ups.

Newer Bracket System

It is the brackets that carry orthodontic force to the teeth during treatment. Despite the fact that metal brackets are better in terms of performance, they are aesthetically less appealing than their plastic counterparts. Brackets are being constructed from tooth-colored materials like porcelain and plastics because of the growing concern about appearance [39].

Ceramic Brackets

A reasonable price can be paid for sections of alumina that have structures consisting of either polycrystalline or single-gem structures. Ceramic brackets have a number of benefits, including a high level of aesthetic appeal, a low level of water absorption, increased mechanical properties, and biocompatibility. Because of the stress caused by the archwire, the bracket wing fractured while tying the ligature. Wear on the teeth at the point of debonding, which causes cracks in the enamel, occurs during the course of therapy [40].

Plastic Brackets

Patients choose polycarbonate plastic braces because they are beautiful to the eye and are made from durable material. Plastic brackets have a number of drawbacks, including an excessive amount of creep deformation, poor torque capacity, a decrease in toughness and longevity, mucosal bruising, and spotty color.

Self-Ligating Brackets

Bracket systems with a mechanical mechanism to seal the edgewise slot are known as self-ligating brackets. As a result, the archwire is held in place in the bracket slot by a cover that is integrated into the bracket itself. The fourth wall of the self-ligating bracket may be moved to transform the slot into a tube [41]. A low-friction, low-force delivery method is the theory underlying this device, ensuring more physiologic tooth movement and a more balanced oral interaction [41]. Active and passive self-ligating bracket systems are the two varieties available. Their interaction with the archwire is referred to by this term. Spring clips on the labial/buccal side of the bracket slot push on the archwire of the self-ligating brackets. The archwire is held securely in place by this mechanism. These include In-Ovation (GAC International, Islandia, NY), SPEED (Strite Industries, Cambridge, Canada), and Time Bracket (Adenta, Munich Germany. The archwire is encased in a robust door or latch, allowing for greater space for the archwire and preventing the clip from pressing on the archwire. The Damon (Ormco, Orange, CA), SmartClipTM (3M Unitek, Irwindale, CA), and Oyster ESL (Gestenco International, Gothenburg, Sweden) are just a few examples. Self-ligating brackets reduce friction between the archwire and the bracket, reduce clinical forces, shorten treatment times, and straighten teeth more quickly.

Apps Used

A new kind of orthodontic software, known as an "app," has also been developed. Apps for diagnosis, treatment planning, communication, and interaction with patients have been developed for iOS and Android. There are a variety of patient-facing apps in this category, such as diagnostics, reminders, and progress tracking, as well as public awareness and education apps, such as peer-reviewed publications and model analysis applications (Table 1) [42].

**Table 1:** Apps used in orthodontics

Clinician apps	Patient apps	Educational apps
Orthodontic Update (provides access to the publications)	BraceMate (provides emergency information to follow if there is any problem, patients can pick colors of the modules they wanted for their teeth)	Glossary of Orthodontic Terms (dictionary app for students to clear concepts of all the terms used in orthodontics)
Doctor Smile Orthodontics (for giving patients education and motivation)	Brace Reminder (notification reminder for tightening)	AJODO (abstracts of articles can be read)
Dolphin MyOrthodontist (helps to connect with the patients, appointments, account balances, and media for patient education can be managed)	My Orthodontist (provides information about orthodontists, FAQs, office hours, and directions)	OneCeph (for cephalometric analysis)
Dental Monitoring (allows remote monitoring of a patient and educates the patient to take good pictures of the teeth.)	Orthodontic Guide (provides information about orthodontic specialty and treatment options)	iModel Analysis (for study model analysis)
REM Orthodontics (for shopping for orthodontic materials)	Trayminder Aligner Tracker (helps the patient to track aligner wear time on each day, shows a reminder to switch to the next aligner, and takes teeth selfies to document progress)	Interceptive Orthodontics (provides step by step guide to early intervention in cases of ectopic eruption of maxillary canines and molars)

Nanotechnology in Orthodontics

Nano is a Greek word that means "dwarf." There are about 109 nanometers in one square inch. Nanotechnology has several uses in dentistry, medicine, and orthodontics, among others. Various applications of nanotechnology in orthodontics include [43] nanocoatings in archwires; using nanoparticles as dry lubricants, orthodontic wires, and brackets are less likely to chafe against one other. For orthodontic stainless steel wires, self-lubricating coatings made of tungsten sulfide (IF-WS2) nanoparticles have been employed. Polymer nanocomposites include silica nanofillers with a diameter of 0.005 to 0.01 microns, which are used in orthodontic adhesives. An enhanced filler load may be accomplished due to the reduction in particle dimensions and the broad dispersion of the particle sizes, which results in lower polymerization shrinkage and improved mechanical characteristics such as tensile, compressive, and break resistance.

Nanoparticle Delivery From Elastomeric Ligature

They may be anti-cariogenic (fluoride), anti-inflammatory, and anti-bacterial medicinal molecules incorporated in the elastomeric matrix of the nanoparticles of the ligatures. Orthodontic wires made from shape-memory nanocomposite polymers may be used to create a more aesthetic orthodontic wire. Clear, colorable, and stain-resistant shape memory polymers (SMP) provide more visually pleasing treatment equipment for patients to enjoy. Oral biofilm control during orthodontic therapy is important. Metal nanoparticles with a diameter of 1-10 nm exhibit bactericidal properties. Dental materials may be treated with nanoparticles or coated with nanoparticles to help limit microbial adhesion to minimize the formation of biofilms. Antibacterial characteristics have been observed for resin composites incorporating silver ion-implanted fillers that release silver ions [43]. In the form of nitrogen-doped titanium dioxide nanoparticles, silver (Ag), gold (Au), silica (SiO2), Cu/CuO, and ZnO nanoparticles are used as antibacterial agents (TiO2).

Smart Brackets With Nanomechanical Sensors

Nanomechanical sensors may be made and placed into the base of orthodontic brackets to give information on how much force is being applied to the tooth by the bracket. A compact, low-profile modern bracket system may be employed to encase a nanochip. Biocompatible coatings, such as titanium nanotubes, may promote early osseointegration and act as an interfacial layer between the newly produced bone and the temporary anchorage devices (TAD) [44].

Microsensor Technology

In addition, a novel microsensor technology has been developed to assist monitor the wear of detachable appliances. Microprocessors the size of a penny are used in this device, which may be removed for cleaning. Using a wireless connection to a computer program, the patient's wear of the device may be calculated.

Digital Study Models

In orthodontic diagnosis and treatment planning, study models are an essential aspect of the process. A record of malocclusion before therapy, treatment phases, and treatment outcomes is also provided by study models. There are a variety of constraints to orthodontists' study models, such as their storage, durability, and transportability. Keeping patient records for at least 11 years is mandated by the 1987 Consumer Protection Act. Plaster study models have been replaced with digital ones thanks to advances in computer technology. For instance, with these digital research models, a laboratory procedure is not required. Furthermore, they require a small amount of storage space, can be retrieved quickly and effectively from any location, are not susceptible to damage, and can be moved around with ease. Other drawbacks of digital study models include the absence of tactile input, high cost, inability to install on an articulator, and the need for extra equipment and technical know-how to use them. For these reasons, orthodontic practitioners were not relying on them to the full extent [44]. Using these estimations, such as teeth's size, curve length, broadness, midline error, space inspection, overjet, Bolton, overbite, molar-canine association, and molar-canine connection, in clinical practice is acceptable [45]. An alginate engraver or intraoral scanner may produce sophisticated models.

## Conclusions

In the last several decades, orthodontics has taken a huge leap toward digital technology. Orthodontists have greatly benefited from the digital age in terms of increasing their productivity and reducing labor costs. Every day, more technologies are found that assist the practitioner in better grasping the issues of the patient, diagnosis, treatment planning, and implementation of the plan for more accurate outcomes. Orthodontists, as well as the rest of the dental profession, have benefited from the aforementioned technical improvements. All areas of dentistry may benefit from these developments (prosthodontics, endodontics, oral surgery, etc.). Software programs have made diagnosis and treatment planning more accurate, simpler, and less time-consuming. Every day, scientists and engineers work tirelessly to make dentistry simpler for dentists all around the globe.
